# High-Intensity Focused Ultrasound Ablation for Unresectable Primary and Metastatic Liver Cancer: Real-World Research in a Chinese Tertiary Center With 275 Cases

**DOI:** 10.3389/fonc.2020.519164

**Published:** 2020-10-29

**Authors:** Yongshuo Ji, Junqiu Zhu, Linglin Zhu, Yanfei Zhu, Hong Zhao

**Affiliations:** HIFU Center of Oncology Department, Huadong Hospital Affiliated to Fudan University, Shanghai, China

**Keywords:** HIFU, hepatocellular carcinoma, metastatic hepatic carcinoma, response, pain, survival, biomakers

## Abstract

This retrospective analysis was conducted to evaluate the feasibility and safety of high-intensity focused ultrasound ablation for primary liver cancer and metastatic liver cancer. Patients with liver cancer who received high-intensity focused ultrasound were included in this analysis, including a primary liver cancer cohort (*n*=80) and a metastatic liver cancer cohort (*n*=195). The primary endpoint of our research was tumor response. The secondary endpoints included survival outcomes, visual analog scale pain scores, alpha-fetoprotein relief, and complications. Objective response rate and disease control rate were observed to be 71.8% and 81.2%, respectively, in patients with primary liver cancer and were 63.7% and 83.2% in cases with metastatic liver cancer. Alpha-fetoprotein levels and visual analogue scale levels significantly decreased after treatment compared with the baseline levels in patients with primary liver cancer (*p*<0.05). Median overall survival was estimated to be 13.0 and 12.0 months in the primary liver cancer and metastatic liver cancer cohorts. The 1-year survival rate was 70.69% and 48.00%, respectively. Multivariate regression analysis showed that visual analogue scale ≥ 5, longest diameter ≥ 5 cm, and portal vein invasion were the independent risk factors for poor survival in primary liver cancer. For patients with metastatic liver cancer, independent risk factors were identified as visual analogue scale ≥ 5, longest diameter ≥ 5 cm, existence of extrahepatic metastases, existence of portal vein invasion, and time to high-intensity focused ultrasound treatment from diagnosis < 3 months. Severe adverse events were rarely reported. In conclusion, high-intensity focused ultrasound might be an effective and safe option for patients with liver cancer regardless of primary and metastatic lesions.

## Introduction

Liver cancer is one of the most common diseases worldwide (1, 2) with estimated deaths ranking fourth among all kinds of cancers (1). Hepatocellular carcinoma (HCC) accounts for approximately 90% of primary liver cancer with steadily rising incidence globally (3, 4). The majority of diagnosed cases of HCC have advanced disease (5), which might cause the poor prognosis. The disease often presents in the setting of advanced cirrhosis, and orthotropic liver transplant provides the greatest chance for both cure and long-term survival (6, 7).

Metastatic liver cancer (MLC) is more prevalent than primary liver cancer (PLC) but no less harmful (8, 9). Liver metastases commonly arise from gastrointestinal cancers, including those of the esophagus, stomach, pancreas, and colorectum, as well as from other solid tumors (10). It is reported that liver metastasis accounts for about 25% of all metastatic disease (11).

The local treatment of hepatic metastases is based on surgical interventions. Surgery is the first choice for the treatment of both primary and secondary liver cancer. However, it is feasible in only 20%–30% of cases (12). Local, nonsurgical options for liver cancer treatment include radiofrequency ablation, transarterial radioembolization and chemoembolization, electroporation, cryotherapy, laser therapy, and various radiotherapy methods (13, 14). Nevertheless, these techniques have multiple limitations (i.e., a traumatic puncture of the parenchyma, the limited size of lesions, and an inability to real-time monitor during the treatment) (15, 16).

High-intensity-focused-ultrasound (HIFU) is an emerging, noninvasive ablation procedure that can ablate various solid tumors, including primary and secondary liver cancer. It can focus ultrasound energy on the lesions of interest and induce tumor coagulative necrosis by thermal effect (17). The promising efficacy of HIFU for HCC has been demonstrated by numbers of studies in spite of high heterogeneity between studies (18). HIFU monotherapy (19–33) could achieve great tumor response with 1-year survival rates of approximately 80%. When combined with transarterial chemoembolization (TACE) (34–42), the results were roughly equal to that of HIFU monotherapy. HIFU local therapy for MLC patients has also been evaluated by several clinical trials (20, 24, 43–47). The response rate was also high—up to about 80%—with the survival outcome remaining unknown. However, these studies included very small sample sizes, and a considerable number of studies are even fewer than 10 patients. We have previously reported the efficacy of HIFU in the treatment of PLC (48) without the results of MLC. However, some limitations of our previous research, such as earlier enrollment with a small sample size and simplicity of the results, prompted us to update the results of HIFU treatment for PLC cases.

We conducted this retrospective analysis with a relatively large sample size to reveal the real-world clinical benefit and safety of HIFU treatment for PLC and MLC cases. Our focus was to determine the response status after HIFU treatment in patients with PLC and MLC. For response evaluation by HIFU ablation, we compared response evaluation criteria in solid tumors (RECIST) 1.1 and modified RECIST (mRECIST) criteria. We also endeavored to determine which factors could affect the response status and survival outcomes. We report results of primary endpoints of objective response rate (ORR) and disease control rate (DCR) as well as secondary outcomes, including overall survival (OS) with 1-year survival rates, pain relief rate, alpha-fetoprotein (AFP) response rate, and adverse events (AEs).

## Materials and Methods

### Patients

Two hundred seventy-five patients who were histologically confirmed with unresectable HCC or MLC in our center from January 2013 to August 2018 were consecutively included in this retrospective analysis. The diagnosis of HCC or MLC is pathologically proven, and the diagnosis of HCC is made by pathology/cytology or according to the American Association for the Study of Liver Diseases (AASLD) (2010) diagnostic criteria (49). The eligibility criteria were as follows: (1) 18 years of age or older; (2) adequate organ function (white blood cell ≥3.9×10^9^/L, absolute neutrophil count ≥1.5×10^9^L, platelets ≥100×10^9^/L, bilirubin ≤2mg/dL; hemoglobin ≥10g/dL, and serum creatinine ≤150mmol/L); (3) Child-Pugh class A-B; (4) life expectancy of ≥ 3 months; (5) prior liver resection, radiotherapy, or chemotherapy allowed; and (6) received at least one post-HIFU response evaluation. The exclusion criteria were as follows: (1) patients who are indicated for liver resection, (2) women with pregnancy or lactation, (3) patients with a previous cerebrovascular event and active infectious disease, (4) patients with clinically significant liver failure (i.e., encephalopathy or ascites found clinically), and (5) patients who received thrombolytic therapy or other anticoagulant therapy within 4 weeks.

This study was approved by the ethics committee of Huadong Hospital and was done following the Declaration of Helsinki. All patients signed a document of informed consent.

### HIFU Treatment

**Figure 1:**
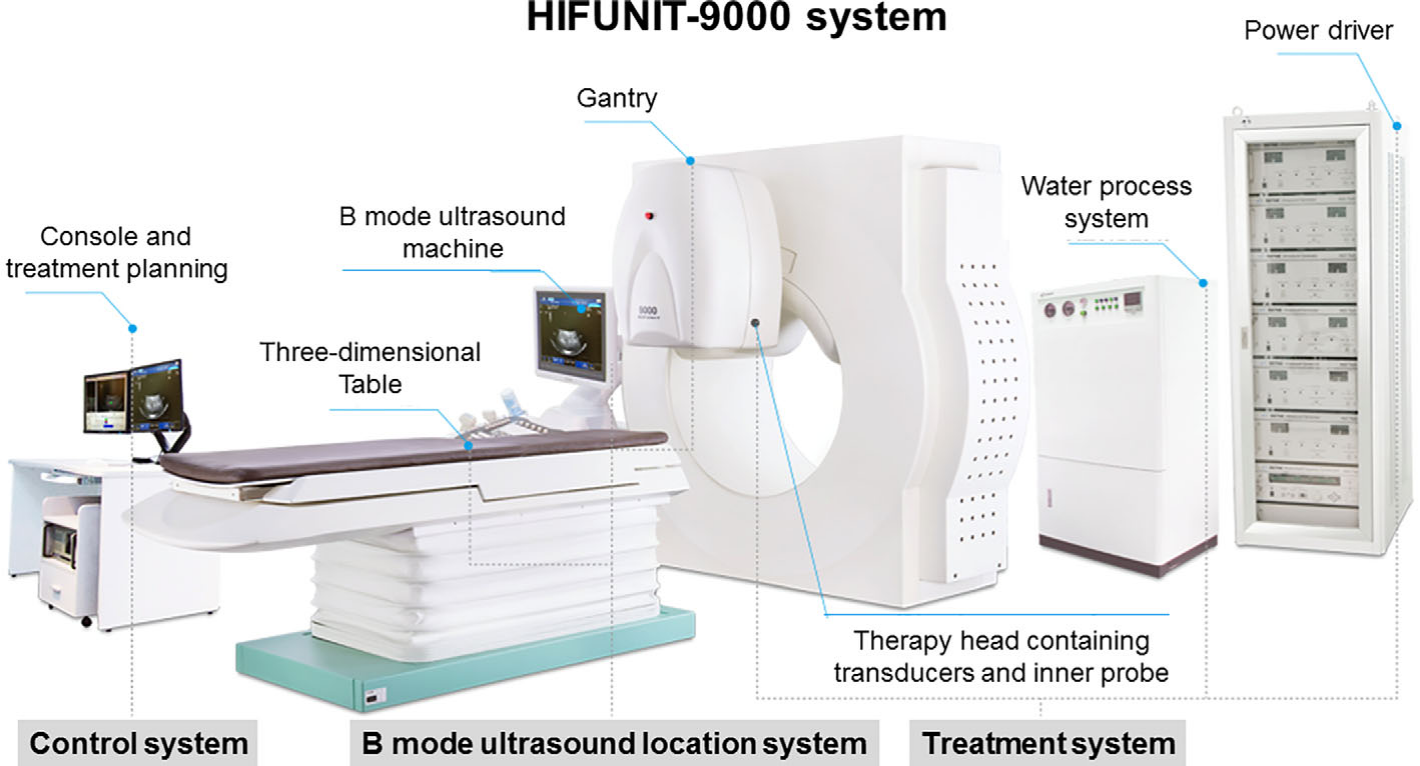
HIFUINT-9000 system. This system consists of three parts: a firing system located in a tank filled with degassed water, an imaging system consisting of an ultrasound scanner coupled with a stereotaxic localizing arm, and a computer that controls the firing sequence and the movement of the firing head through a three-dimensional micropositioning system.

HIFU ablation was performed using the HIFUINT-9000 system (Shanghai A&S Sci-Tec Co., LTD, Shanghai, China), which is a B-mode ultrasound-guided device (**Figure 1**) (50). The patient was fasted to gas-producing food 3 days before treatment and banned water in the morning of the treatment day. During treatment, the patient was placed in the supine position without antibiotics or anesthesia. First, the tumor location, size, and morphological characteristics were identified by computed tomography (CT), b-mode sonography, or magnetic resonance imaging (MRI). Meanwhile, the influence of the tumor on adjacent organs and blood vessels was also evaluated. Next, the detecting head of this system completed the relocalization of the therapy area. Finally, the ablation energy focus was controlled to move along with a three-dimensional axis until it covered the target lesions. The input power was 5–10 kW/cm^2^, and therapy depth was 2–15 cm. The practice-focused sphere was 3 x 3 x 8 mm. The unit ultrasonic pulse included transmit time of 200 ms and intermission time of 400 ms, respectively. Each focused sphere needs the ultrasonic pulse 8–10 times. The average treatment and sonication time was about 30 minutes and 10 minutes, respectively. All of the parameters were allowed to be varied depending on the depth of the tumor.

### Observation and Measurement

The primary endpoints were the ORR with duration of response (DOR) and DCR in the overall cohort and PLC and MLC, respectively. The secondary endpoints were OS with 1-year survival rates, pain relief rate, AFP response rate, and safety. The assessments of hepatic lesions were done with a CT or MRI scan at baseline, 1 month, 3 months, 6 months, and 12 months after HIFU ablation. The RECIST 1.1 (51) and mRECIST (52) were separately used to assess the tumor response at 1 month after HIFU treatment and followed at 2 months. ORR = [(complete response (CR) + partial response (PR)]/total x 100%, and DCR = [(CR+PR +stable disease (SD)]/total x 100%. Visual analogue scale (VAS) value was used as the indicator of pain. HIFU-related AEs were recorded, and the severity was graded by the CTCAE, version 4 (53).

### Statistical Analysis

All the data analyses were conducted using the statistical software of STATA Version 11.0 (College Station, TX, USA). Our data were described as the mean ± SD for normally distributed data or median with range for non-normally distributed data. OS analysis of patients was conducted by the Kaplan-Meier method. Potential independent risk factors for survival were evaluated by univariate analysis (log-rank test) and multivariate analysis (Cox proportional hazards model). *P*-value < 0.05 indicated statistical significance.

## Results

### Baseline Characteristics

According to the inclusion criteria, 275 patients were included finally, including 85 cases with HCC and 190 cases with MLC. The baseline characteristics of these cases are listed in **Table 1**. The cohort of HCC consisted of 54 and 31 cases with Stage III and Stage IV, in which 19 cases (22.4%) had intrahepatic metastasis. For patients with MLC, the common primary tumor sites were colon (*n*=43, 22.6%), pancreas (*n*=42, 22.1%), stomach (*n*=29, 15.3%), rectum (*n*=17, 8.9%), breast (*n*=17, 8.9%), gallbladder (*n*=10, 5.3%), and others, which were fewer than 10 cases. There were 58 (30.5%) that were synchronous (diagnosed concomitantly or within 3 months of the primary tumor) and 132 (69.5%) that were metachronous, in which 52 cases (27.4%) were diagnosed beyond 1 year of the former tumor. AFP levels of HCC and MLC groups were 639.3 ± 106.8 μg/L and 182.7 ± 37.2 μg/L with positive rates of 77.6% and 13.2%, respectively. Histories of hepatitis B (45.9% vs. 3.7%) and liver cirrhosis (22.4% vs. 0.7%) were more common in patients with HCC compared with patients with MLC. VAS ≥ 5 was also more commonly seen (40% vs. 6.3%) in the HCC group than the MLC group.

**Table 1:** Baseline characteristics of patients and tumors.

Characteristics	PLCn=85	MLCn=190	OverallN=275
**Age**			
** Median (range), year** ** <65, n (%)** ** ≥65, n (%)**	63 (37-89) 48 (56.5) 37 (43.5)	63 (31-89) 102 (53.7) 88 (46.3)	63 (31-89) 150 (54.5) 125 (45.5)
**Gender**			
** Male, n (%)** ** Female, n (%)**	69 (81.2) 16 (18.8)	111 (58.4) 79 (41.6)	180 (65.5) 95 (34.5)
**ECOG PS**			
** Median (range)** ** <2, n (%)** ** ≥2, n (%)**	2 (0-4) 46 (54.1) 39 (45.9)	2 (0-4) 77 (40.5) 113 (59.5)	2 (0-4) 123 (44.7) 152 (55.3)
**VAS**			
** Median (range)** ** < 5, n (%)** ** ≥ 5, n (%)**	5 (0-10) 51 (60.0) 34 (40.0)	1 (0-7) 178 (93.7) 12 (6.3)	3 (0-10) 229 (83.3) 46 (16.7)
**Lesions number**			
** Median (range)** ** Single, n (%)** ** Multiple, n (%)**	3 (1-6) 16 (18.8) 69 (81.2)	3 (1-7) 29 (15.3) 161 (84.7)	3 (1-7) 45 (16.4) 230 (83.6)
LDi*			
** Mean ( ± SE), cm** ** < 5 cm, n (%)** ** ≥ 5 cm, n (%)**	4.94 ( ± 1.67) 51 (60.0) 34 (40.0)	5.37 ( ± 1.90) 87 (45.8) 103 (54.2)	138 (50.2) 137 (49.8)
**Portal vein invasion**			
** Yes, n (%)** ** No, n (%)**	33 (38.8) 52 (61.2)	44 (23.2) 146 (76.8)	77 (28.0) 198 (72.0)
**Tumor location**			
** Right lobe, n (%)** ** eft lobe, n (%)** ** Both, n (%)**	7 (8.2) 18 (21.2) 60 (70.6)	14 (7.4) 24 (12.6) 152 (80.0)	21 (7.6) 42 (15.3) 212 (77.1)
**Stage of PLC**		NA	NA
** Stage III** ** Stage IV**	54 (63.5) 31 (36.5)		
**Intrahepatic metastasis status**		NA	NA
** Yes, n (%)** ** No, n (%)**	19 (22.4) 66 (77.6)		
**Extrahepatic metastases status**			
** Yes, n (%)** ** No, n (%)**	22 (25.9) 63 (74.1)	70 (36.8) 120 (63.2)	92 (33.5) 183 (66.5)
**Liver metastases present at initial diagnosis**	NA		NA
** Median time^&^, month** **(range)** ** Yes (synchronous), n (%)** ** No (metachronous), n (%)** ** < 12 months** ** ≥ 12 months**		6 (0-132) 58 (30.5) 132 (69.5) 80 (42.1) 52 (27.4)	
**Primary tumor site for MLC**	NA		NA
** Colon, n (%)** ** Pancreas, n (%)** ** Gastric, n (%)** ** Rectum, n (%)** ** Breast, n (%)** ** Gallbladder, n(%)** ** Others^☨^**		43 (22.6) 42 (22.1) 29 (15.3) 17 (8.9) 17 (8.9) 10 (5.2) 32 (16.8)	
**Histories of disease**			
** Hepatitis B, n (%)** ** Liver cirrhosis, n (%)** ** Hypertension, n (%)** ** Diabetes Mellitus, n (%)**	39 (45.9) 19 (22.4) 17 (20.0) 10 (11.8)	7 (3.7) 1 (0.5) 29 (15.3) 17 (8.9)	46 (16.7) 20 (7.3) 46 (16.7) 27 (9.8)
**Prior therapies for liver cancer**			
** Median (range)** ** Prior ≥one therapies, n (%)** ** Prior surgery, n (%)** ** Prior RFA, n(%)** ** Prior TACE, n (%)** ** Prior chemotherapy, n (%)**	1 (0-3) 14 (16.5) 13 (15.9) 2 (2.4) 1 (1.2) 1 (1.2)	0 (0-3) 6 (3.2) 5 (2.6) 1 (0.5) 0 1 (0.5)	0 (1-3) 20 (7.3) 18 (6.5) 3 (1.1) 1 (0.4) 2 (0.7)
**AFP level**			
** Mean (± SE), μg/L** ** Negative (< 400 μg/L), n (%)** ** Positive (≥ 400 μg/L), n (%)**	639.3 (± 106.8) 19 (22.4) 66 (77.6)	182.7 ( ± 37.2) 165 (86.8) 25 (13.2)	323.83 (± 71.3) 184 (66.9) 91 (33.1)
**Time to HIFU^#^**			
** Median (range), months** ** < 3, n (%)** ** ≥ 3, n (%)**	3 (0.5-83) 40 (47.1) 45 (52.9)	3 (0.5-24) 93 (48.9) 97 (51.1)	3 (0.5-83) 133 (48.4) 142 (51.6)
**HIFU sessions**			
** Median (range), months** ** < 5, n (%)** ** ≥ 5, n (%)**	5 (2-40) 14 (16.5) 71 (83.5)	5 (2-40) 47 (24.7) 143 (75.3)	5 (2-40) 61 (22.2) 214 (77.8)
**Indicated for RFA**			
** Yes, n (%)** ** No, n (%)**	40 (47.1) 45 (52.9)	81 (42.6) 109 (57.4)	121 (44.0) 154 (56.0)

* LDis for the patients with multiple lesions were the sum of the longest diameter of all these lesions.

^#^Time to HIFU ablation from the diagnosis of HCC.

^&^Median time of liver metastases from initial diagnosis.

^☨^Others included lung cancer (n=8), esophagus cancer (n=5), renal cancer (n=3), nasopharynx cancer (n=3), melanoma (n=3), cervical cancer (n=2), bladder cancer (n=2), ovarian cancer (n=1), prostatic cancer (n=1), skin cancer (n=1), unknown (n=3).

AFP, alpha-fetoprotein; ECOG PS, Eastern Cooperative Oncology Group Performance Status; HCC, Hepatocellular Carcinoma; HIFU, High-Intensity Focused Ultrasound; PLC, primary liver cancer; RFA, radiofrequency ablation; LDi, longest diameter; MLC, metastatic liver cancer; NA, not applied; TACE, transarterial chemoembolization; VAS, visual analogue scale.

### ORR and DCR

All 275 cases received a response evaluation by mRECIST and RECIST 1.1 criteria, respectively. The CR rate, ORR, and DCR were estimated to be 53.8% (95% CI = 47.9–59.7%), 66.2% (95% CI = 60.6–71.8%), and 82.5% (95% CI = 78.0–87.1%) by mRECIST. However, when RECIST 1.1 was used, no CR was identified, and ORR and DCR were 31.3% (95% CI = 23.3–38.7%) and 71.6% (95% CI = 64.2–77.9%), respectively, which were also significantly lower than the rates by mRECIST (*p*=0.001). The results were described as PLC and MLC separately with subgroup analyses (**Table 2**).

**Table 2:** Objective response rate (ORR) and disease control rate (DCR) and subgroup analyses.

Response	mRECIST criteria, CR/ORR/DCR		RECIST1.1 criteria, ORR/DCR	
	PLC, n=85	MLC, n=190	PLC, n=85	MLC, n=190
**Total, %**	63.5/71.8/81.2	49.5/63.7/83.2	32.9/74.1	30.5/70.5
**Age** ** <65, %** ** ≥65, %**	P=0.790 62.5/72.9/83.3 64.9/70.3/78.4	P=0.890 48.0/62.7/81.4 51.1/64.8/85.2	P=0.433 33.3/79.2 32.4/67.6	P=0.418 34.3/73.5 26.1/67.0
**Gender** ** Male, %** ** Female, %**	P=0.770 60.9/69.4/79.7 75.0/81.3/87.5	P=0.283 45.0/63.1/82.9 55.7/64.6/83.5	P=0.762 31.8/72.5 37.5/81.3	P=0.396 34.2/73.0 25.3/67.1
**ECOG PS** ** <2, %** ** ≥2, %**	P=0.019 71.7/84.8/89.1 53.8/56.4/71.8	P=0.006 48.1/71.4/90.9 50.4/58.4/77.9)	P=0.000 52.2/84.8 10.3/61.5	P=0.001 44.2/83.1 21.2/61.9
**VAS** ** < 5, %** ** ≥ 5, %**	P=0.017 68.6/82.4/88.2 55.9/55.9/70.6	P=0.415 50.6/64.6/84.3 33.3/50.0/66.7	P=0.000 49.0/86.3 8.8/55.9	P=0.632 30.9/71.3 25.0/58.3
**Lesions number** ** Single, %** ** Multiple, %**	P=0.766 68.8/81.3/87.5 62.3/69.6/79.7	P=0.900 48.3/62.1/86.2 49.7/64.0/82.6	P=0.079 56.3/87.5 27.5/71.0	0.385 41.4/75.9 28.6/69.6
**LDi^&^** ** < 5 cm, %** ** ≥ 5 cm, %**	P=0.003 74.5/84.3/94.1 47.1/52.9/61.8	P=0.010 56.3/75.9/89.7 43.7/53.4/77.7	P=0.000 43.1/90.2 17.6/50.5	P=0.010 41.4/77.0 21.4/65.0
**Portal vein invasion** ** Yes, %** ** No, %**	P=0.001 45.5/51.5/60.6 75.0/84.6/94.3	P=0.000 29.5/47.7/56.8 55.5/68.5/91.1	P=0.003 31.7/57.5 36.3/81.2	P=0.007 24.5/66.9 40.5/75.7
**Tumor location** ** Right lobe, %** ** Left lobe, %** ** Both, %**	P=0.056 57.1/71.4/71.4 72.2/94.4/100.0 61.6/65.0/76.7	P=0.388 35.7/57.2/78.6 62.5/83.3/95.8 48.6/61.2/81.6	P=0.093 33.2/69.8 45.9/86.5 34.8/72.0	P=0.120 27.9/66.7 38.6/74.9 29.6/64.2
**Stage of PLC** ** Stage III, %** ** Stage IV, %**	P=0.270 70.4/77.8/87.0 51.6/61.3/71.0	NA	P=0.189 38.9/79.6 22.6/64.5	NA
**Intrahepatic metastasis status** ** Yes, %** ** No, %**	P=0.881 63.2/73.7/78.9 63.6/71.2/81.8	NA	P=0.844 36.8/78.9 31.8/72.7	NA
**Extrahepatic metastases status** ** Yes, %** ** No, %**	P=0.574 59.1/68.2/72.7 65.1/73.0/84.1	P=0.001 42.9/47.1/88.3 53.3/73.3/74.3	P=0.424 27.3/63.6 34.9/77.8	P=0.001 14.3/67.1 40.0/72.5
**Liver metastases present at initial diagnosis** **Synchronous, %** ** Metachronous(< 1 year), %** ** Metachronous (≥ 1 year), %**	NA	P=0.340 51.7/62.1/81.0 52.5/71.3/85.0 42.3/53.8/82.7	NA	P=0.370 25.8/62.1 36.3/75.0 26.9/73.1
**Primary tumor site for MLC** ** Colon, %** ** Pancreas, %** ** Gastric, %** ** Rectum, %** ** Breast, %** ** Gallbladder, %** ** Others^☨^, %**	NA	P=0.013 58.1/74.4/90.7 42.9/50.0/73.8 34.5/55.2/82.8 47.1/64.7/95.1 70.6/.76.5/82.4 70.0/70.0/90.0 43.8/65.6/78.1	NA	P=0.008 39.5/83.7 9.5/52.4 41.4/79.3 35.3/88.2 23.5/52.9 10.0/60.0 43.8/71.9
**Histories of disease** ** Hepatitis B, %** ** Liver cirrhosis, %** ** Hypertension, %** ** Diabetes Mellitus, %**	P=0.593 69.2/76.9/84.6 68.4/78.9/84.2 82.4/82.4/88.2 70.0/80.0/90.0	P=0.477 71.4/85.7/85.7 100/100/100 44.8/69.0/86.2 58.8/70.6/88.2	P=0.794 35.9/82.1 31.6/78.9 29.4/70.6 40.0/80.0	P=0.652 28.6/85.7 31.6/78.9 37.9/72.4 23.5/64.7
**Prior therapies for liver cancer** ** Prior ≥one therapies, %** ** Naïve, %**	P=0.236 57.1/64.3/64.3 64.8/73.2/84.5	P=0.097 100/100/100 47.8/62.5/82.6	P=0.519 35.7/64.3 34.2/76.1	P=0.944 33.3/66.7 30.4/70.7
**AFP level** ** Negative (< 400 μg/L), %** ** Positive (≥ 400 μg/L), %**	P=0.464 73.7/73.7/84.2 60.1/71.2/80.3	P=0.922 49.1/63.6/83.6 52.0/64.0/80.0	P=0.111 52.6/84.2 27.3/71.2	P=811 29.7/70.3 36.0/72.0
**Time to HIFU^#^** ** < 3 months, %** ** ≥ 3 months, %**	P=0.826 62.5/70.0/82.5 64.4/73.3/80.0	P=0.371 55.9/68.8/84.9 43.3/58.8/81.4	P=0.488 27.5/75.0 37.8/73.3	P=0.523 34.4/72.0 26.9/69.1
**HIFU sessions** ** < 5, %** ** ≥ 5, %**	P=0.509 64.3/64.3/71.4 63.4/73.2/83.1	P=0.108 38.3/53.2/76.6 53.1/67.1/85.3	P=0.519 21.4/64.3 35.2/76.1	0.042 19.1/57.4 34.3/74.8
**Indicated for RFA** ** Yes, %** ** No, %**	P=0.443 70.0/77.5/87.5 57.7/68.9/75.5	P=0.340 53.1/70.4/87.7 46.8/58.7/79.8	P=0.279 35.1/76.0 30.5/65.9	P=0.197 33.2/73.9 27.8/67.4

All the p-values were from chi-square tests based on the number of patients who achieved CR, PR, SD, and PD in each subgroup.

^&^LDis for the patients with multiple lesions were the sum of the longest diameter of all these lesions.

^#^Time to HIFU ablation from the diagnosis of PLC or MLC.

^☨^Others included lung cancer(n=8), esophagus cancer (n=5), renal cancer (n=3), nasopharynx cancer (n=3), melanoma (n=3), cervical cancer (n=2), bladder cancer (n=2), ovarian cancer (n=1), prostatic cancer (n=1), skin cancer (n=1), unknown (n=3).

AFP, alpha-fetoprotein; CR, complete response; DCR, disease control rate; ECOG PS, Eastern Cooperative Oncology Group Performance Status; HIFU, High-Intensity Focused Ultrasound; LDi, longest diameter; ORR, objective response rate; PLC, primary liver cancer; PD, Progressive Disease; MLC, metastatic liver cancer; PR, partial response; RECIST, response evaluation criteria in solid tumors; RFA, radiofrequency ablation; SD, stable disease; VAS, visual analogue scale.

**Figure 2:**
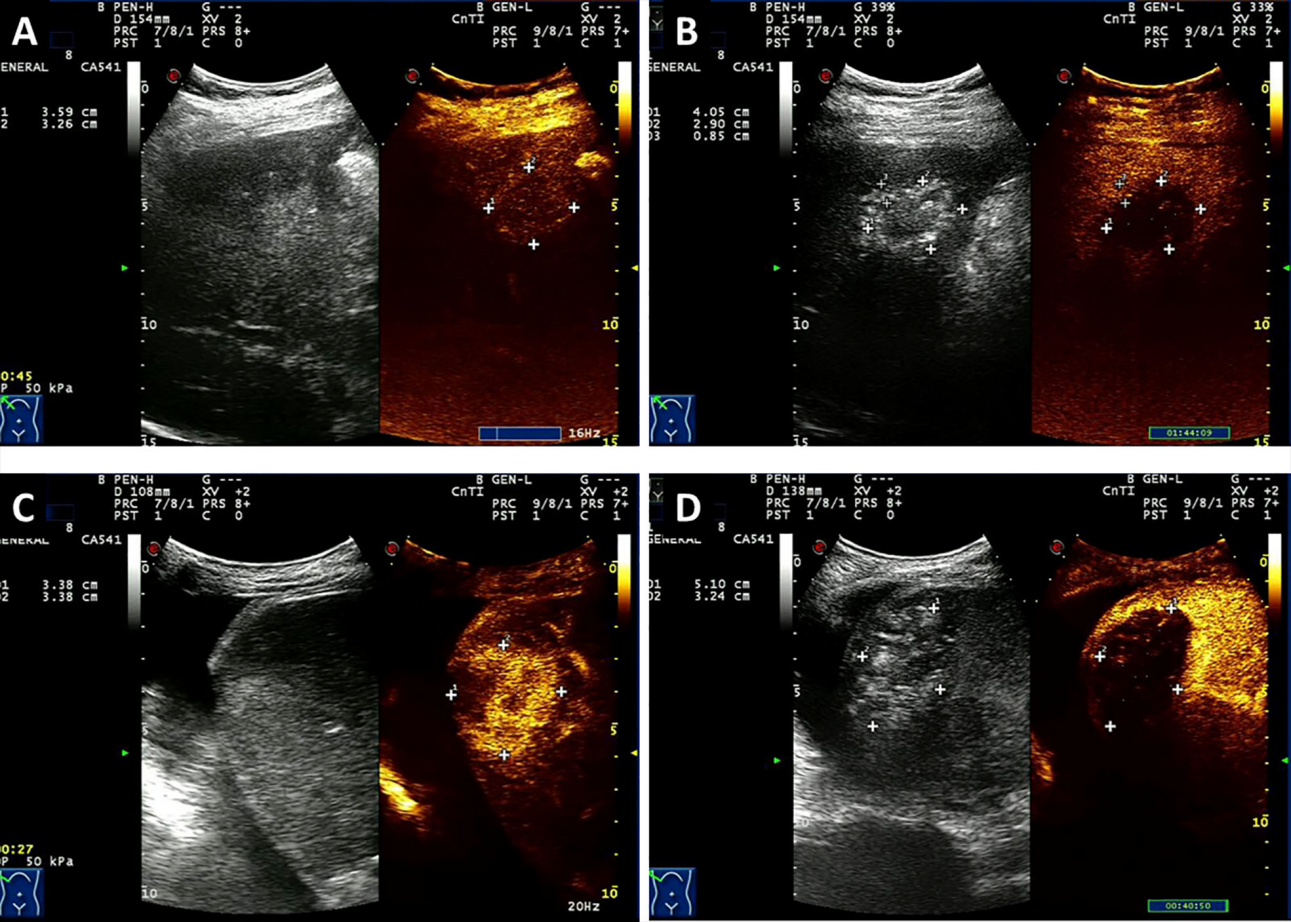
Contrast-enhanced ultrasound (CEUS) of one patient with HCC **(A, B)** and a case with liver metastases from rectal cancer **(C, D)** who was treated with HIFU. A patient was newly diagnosed with stage III HCC by puncture biopsy with AFP level > 1000 U/ml and a previous history of hepatitis B. The patient was treated with HIFU without other therapies. **(A)**. Before HIFU ablation, CEUS depicts the enhancing lesion with a richness of blood supply. **(B)** After treatment, CEUS reveals a completely ablated lesion that shows criteria intratumoral perfusion defect and lacks contrast enhancement. Another patient underwent Dixon surgery and received postsurgery Xelox chemotherapy for six cycles. Twelve months later, hepatic metastatic carcinoma was detected. **(C)** Before HIFU ablation, CEUS depicts the enhancing lesion with a richness of blood supply. **(D)** After HIFU ablation, a intratumoral perfusion defect was seen with no contrast enhancement which indicates complete ablation.

According to the mRECIST criteria, 63.5% (95% CI = 53.1–74.0) of patients with HCC achieved CR after HIFU ablation (**Figure 2**) with an ORR of 71.8% (95% CI = 62.0–81.5) and DCR of 81.2% (95% CI = 72.7–89.7), respectively. The CR rate, ORR, and DCR were 49.5% (95% CI = 42.3–56.6), 63.7% (95% CI = 56.8–70.6), and 83.2% (95% CI = 77.8–88.5), respectively, in patients with MLC.

No CR was observed by RECIST 1.1 in cases with both HCC and MLC. For patients with HCC, 32.9% (95% CI = 22.7–43.1%) of cases were responding to HIFU ablation, and 74.1% (95% CI = 64.6–83.6%) of cases obtained disease control. The ORR and DCR were 30.5% (95% CI = 23.9–37.1%) and 70.5% (95% CI = 64.0–77.1%), respectively, in patients with MLC.

Chi-square tests revealed that the Eastern Cooperative Oncology Group performance status (ECOG PS), VAS, longest diameter (LDi), and portal vein invasion would affect the response outcome in patients with HCC. In patients with ECOG PS < 2, the ORR and DCR were 52.2% (95% CI = 37.4–67.0%) and 84.8% (95% CI = 74.1–95.4%), respectively, which were significantly higher than those with ECOG PS ≥ 2. Similar superiority was shown in the patients with VAS < 5, with LDi < 5 cm, and without portal vein invasion (**Table 2**). In the MCL cohort, cases with hepatic metastasis from colon (ORR = 39.5%, DCR = 83.7%) and rectum (ORR = 35.3%, DCR = 88.2%) experienced better response status. Meanwhile, patients with liver metastasis from pancreas (ORR = 9.5%, DCR = 52.4%) and gallbladder (ORR = 10.0%, DCR = 60.0%) had poor response. In addition, some patient characteristics including ECOG PS ≥ 2, LDi ≥ 5 cm, existence of extrahepatic metastases, existence of portal vein invasion, and HIFU ablation times < 5 would diminish the response rate and control rate.

### Duration of Response

One hundred eighty-two cases were responding to HIFU ablation by mRECIST, of which the median DOR was 9.5 months with a 95% CI of 8.8–10.2 months. The median DOR was 10.8 (95% CI = 9.8–12.2) months and 8.5 (95% CI = 6.8–9.5) months in 61 responders with HCC and 121 responders with MLC, respectively.

**Figure 3:**
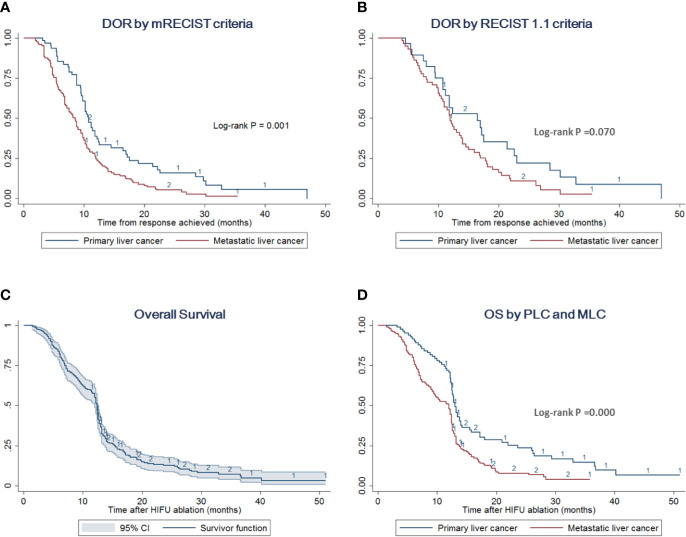
DOR and OS by different liver cancer types. **(A)** DOR stratified analysis by PLC and MLC according to the mRECIST criteria. The log-rank test indicates that patients with PLC prolong the DOR compared with cases with MLC (*p* = 0.001). **(B)** DOR stratified analysis by PLC and MLC according to the RECIST 1.1 criteria. The log-rank test indicates no difference between these two groups (*p* = 0.070) **(C)** OS with 95% CI of the overall cohort including PLC and MLC. **(D)** OS stratified analysis by PLC and MLC. The log-rank test indicates that patients with PLC would improve the survival outcome compared with cases with MLC (*p* = 0.000).

When RECIST 1.1 criteria were used, shown in **Figure 3**, median DOR was calculated to be 12.2 (95% CI = 11.0–14.6) months with a 1-year response rate of 50.98% (95% CI =39.96–60.97%). For 28 responded cases with HCC, DOR was 16.5 (95% CI: 10.8–21.4) months and 1-year response rate was 52.78% (95% CI = 32.84–69.30%). For 58 patients with metastatic liver cancer, in whom a PR was achieved, median DOR was 12.0 months (95% CI = 10.5–13.8). The 1-year response rate was 48.28% (95% CI = 35.00–60.34%). There was no significant difference between these two groups (log-rank *P* value = 0.07).

### AFP and VAS Relief

The relief of AFP levels and VAS scores are presented in **Table 3**. AFP levels were significantly decreased to 361.6 ± 79.3 μg/L in the patients with PLC after HIFU ablation compared with baseline levels of 639.3 ± 106.8 μg/L. An obvious decrease in AFP levels was detected in 43 (50.59%) cases. However, in cases with MLC and the overall cohort, AFP decrease was not significant. Similarly, VAS levels were significantly decreased in the overall cases, especially in the cases with PLC: 38.8% of patients achieved obvious pain relief with VAS reduction of at least 20%, 50%, and 80% in 13 (15.3%), 17 (20.0%), and 3 (3.5%) cases, respectively.

**Table 3:** Alpha-fetoprotein (AFP) and visual analog scale (VAS) relief.

	PLC, n=85	MLC, n=190	Total, n=275
**AFP response**			
** AFP levels, μg/L; mean ± SE**			
**Baseline** **1 month after HIFU**	639.3 ± 106.8 361.6 ± 79.3*	182.7 ± 37.2 155.3 ± 44.6	323.83 ± 71.3 229.13 ± 77.8
**Decrease, n (%)**			
** ≥ 20%** ** ≥ 50%** ** ≥ 80%**	27 (31.8%) 12 (14.1%) 4 (4.7%)	7 (3.7%) 0 1 (0.5%)	34 (12.4%) 12 (4.4%) 5 (1.8%)
**AFP from positive to negative, n (%)**	27/66 (40.9%)	3/25 (12.0%)	30/91 (33.0%)
** VAS relief**			
** VAS levels**			
** Baseline** **1 month after HIFU**	3.894 ± 1.352 2.145 ± 0.892*	1.942 ± 0.736 1.647 ± 0.772	2.545 ± 1.143 1.800 ± 1.274*
**Decrease, n (%)**			
** ≥ 20%** ** ≥ 50%** ** ≥ 80%**	13 (15.3%) 17 (20.0%) 3 (3.5%)	7 (3.7%) 3 (1.6%) 0	20 (7.3%) 20 (7.3%) 3 (1.1%)

*p<0.05 after HIFU ablation vs. baseline.

AFP, alpha-fetoprotein; HIFU, High-Intensity Focused Ultrasound; PLC, primary liver cancer; MLC, metastatic liver cancer; VAS, visual analogue scale.

### OS and Subgroup Analyses

The survival outcomes were recorded in 257 cases out of 275 patients with median follow-up of 18 (range, 2–44) months. The median OS was estimated to be 12.4 (95% CI = 12–12.6) months with a 1-year survival rate of 55.23% (95% CI = 48.94–61.07%) (**Figure 3**). Separately, patients with PLC had a median OS of 13.0 (95% CI = 12.5–14.0) months with a 1-year survival rate of 70.69% (95% CI = 59.54–79.29%). Meanwhile, the median OS of the MLC group was estimated to be 12.0 (95% CI = 9.5–12.4) months with a 1-year survival rate of 48.00% (95% CI = 40.43–55.16%) (**Figure 3**, **Supplementary Tables 1** and **2**).

**Figure 4:**
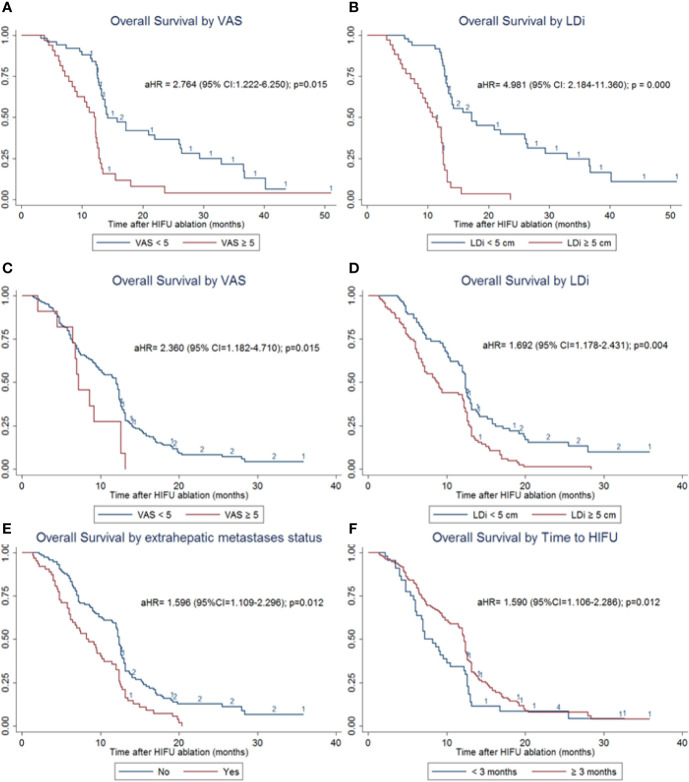
OS subgroup analyses of patients with PLC and MLC. **(A)** OS stratified analysis by VAS in patients with PLC. VAS ≥ 5 significantly increases the risk of death [aHR= 2.784 (95% CI = 1.222–6.250); p = 0.015]. **(B)** OS stratified analysis by LDi in patients with PLC. LDi ≥ 5 cm also significantly increases death risk (aHR = 4.981 (95% CI=2.184–11.360); p = 0.000) **(C)** OS stratified analysis by VAS in patients with MLC. VAS ≥ 5 significantly increases the risk of death (aHR = 2.360 (95% CI=1.182–4.710); p = 0.015). **(D)** OS stratified analysis by LDi in patients with MLC. LDi ≥ 5 cm also significantly increases death risk (aHR = 1.692 (95% CI = 1.178–2.431); p = 0.004). **(E)** OS stratified analysis by extrahepatic metastases status in patients with MLC. Existence of extrahepatic metastases was one of the independent risk factors for poor prognosis (aHR=1.596 (95% CI = 1.109–2.296); p=0.012). **(F)**. OS stratified analysis by the time to HIFU treatment in patients with MLC. Time interval ≥ 3 months was one of the independent risk factors for poor prognosis (aHR=1.590 (95% CI = 1.106–2.286); p = 0.012).

In the subgroup analyses of OS for PLC patients, log-rank tests suggested that factors including ECOG PS, VAS, lesion number, LDi, stage, and AFP levels might influence the survival time. Multivariate regression analysis adjusted with the above factors showed that VAS ≥ 5 [(adjusted Hazard ratio (aHR) = 2.784 (95% CI = 1.222–6.250); *p*=0.015] and LDi ≥ 5 cm (aHR=4.981 (95% CI = 2.184–11.360); *p*=0.000) were the independent risk factors for the poor OS outcome (**Figure 4** and **Supplementary Table 1**).

For patients with MLC, independent risk factors were identified as VAS ≥ 5 (aHR= 2.360 (95% CI = 1.182–4.710); *p*=0.015), LDi ≥ 5 cm (aHR=1.692 (95% CI = 1.178–2.431); *p*=0.004), existence of extrahepatic metastases (aHR=1.596 (95% CI = 1.109–2.296); *p*=0.012), and time to HIFU treatment from diagnosis < 3 months (aHR=1.590 (95% CI = 1.106–2.286); *p*=0.012) (**Figure 4** and **Supplementary Table 2**). These results were adjusted by multiple variables identified in univariate analyses, including gender, ECOG PS, VAS, lesion number, LDi, primary tumor site, extrahepatic metastases status, time to HIFU from diagnosis, and HIFU ablation times.

The correlation between OS with RECIST 1.1 or mRECIST was also compared. The response status evaluated by mRECIST criteria was found to be an independent risk factor for OS in cases with both PCL (aHR=1.725 [95% CI = 1.173–2.639], *p*=0.037) and MCL (aHR=1.350 [95% CI = 1.083–2.350], *p*=0.014). However, the results based on RECIST 1.1 criteria did not reveal any significant correlation with OS.

### HIFU-Related Adverse Events (TEAEs)

Most common TEAEs were body temperature increase (33/275, 12.0%) and abnormal cardiac rhythm (27/275, 9.8%). No skin burns at operation spots occurred. After HIFU ablation, a total of 105 cases (38.2%) reported AEs, of which 51 cases reported more than one complication. Most frequent HIFU-related AEs included fatigue (13.1%), fever (11.3%), abdominal pain (9.8%), rib osteonecrosis injuries (8.7%), diarrhea (5.8%), elevated AST levels (4.0%), elevated ALT levels (3.6%), and rash (2.5%) (**Table 4**). All these complications were mild without the need for symptomatic treatment. Liver abscess and biliary tract obstructions were reported in two patients (0.7%) each. Cases with Liver abscess needed drain placement and antibiotic management. They were discharged from hospital after 17 and 20 days, respectively. Cases with biliary tract obstructions needed stent placement. They were both discharged within a 1-week hospital stay.

**Table 4:** Most Frequent HIFU-related Adverse Events.

HIFU-related AEs	PLC (n=85)	MLC (n=190)	Total (N=275)
**Fatigue**	9 (10.6%)	27 (14.2%)	36 (13.1%)
**Fever**	6 (7.1%)	25 (13.2%)	31 (11.3%)
**Abdominal pain**	8 (9.4%)	19 (10.0%)	27 (9.8%)
**Rib osteonecrotic injures**	7 (8.2%)	17 (8.9%)	24 (8.7%)
**Diarrhea**	6 (7.1%)	10 (5.3%)	16 (5.8%)
**Elevated AST levels**	3 (3.5%)	8 (4.2%)	11 (4.0%)
**Elevated ALT levels**	4 (4.7%)	6 (3.2%)	10 (3.6%)
**Rash**	2 (2.4%)	5 (2.6%)	7 (2.5%)
**Liver abscess**	0	2 (1.1%)	2 (0.7%)
**Biliary tract obstructions**	1 (1.2%)	1 (0.5%)	2 (0.7%)
**Total**	33* (38.8%)	72# (37.9%)	105 (38.2%)

*Thirteen cases with PLC reported more than one AE.

^#^Thirty-eight patients with MLC reported more than one AE.

ALT, alanine aminotransferase; AST, aspartate aminotransferase; HIFU, High-Intensity Focused Ultrasound; MLC, metastatic liver cancer; PLC, primary liver cancer.

## Discussion

Patients with HCC are often unresectable at the time of diagnosis and have a poor long-term prognosis, notwithstanding recent advances in TACE (54). Due to its abundant blood supply, the liver is one of the organs most frequently affected by metastatic disease (55). Although percutaneous ablation techniques have been successfully applied for the treatment of hepatic and renal tumors and are now clinically acceptable alternatives to surgery in selected patients (56), similar procedures are not yet considered by current treatment guidelines for PLC or secondary liver cancer (57–60).

Ablation with HIFU is a noninvasive procedure (61, 62) based on the principle that focused ultrasonic beams cause coagulative necrosis of the target tissue. In addition, HIFU might also improve liver function and enhance the immune function of patients with liver cancer, which could benefit the survival of patients (63). In spite of the clinical potential of HIFU ablation, the liver is a particularly challenging organ for this technique due to the combined effect of respiratory-induced liver motion, partial blocking by the rib cage, and high perfusion/flow (64). In general, the application of HIFU in patients with liver cancer, especially in patients with metastatic liver cancer, is still in the exploratory stage.

We prefer to use CEUS, CT, or MRI and not use the echogenic changes as an indicator of the immediate posttreatment response. The echogenic cloud is not as accurate as contrast-enhanced CT or MR for evaluation of the extent of treatment. The echogenic cloud usually overestimates the extent of ablation, and it is difficult to delineate the exact boundaries of the ablated lesion. In addition, with the long ablation time associated with HIFU, the earlier echogenic changes may not be as conspicuous by the end of the procedure, which limits the use of the echogenic cloud for evaluation of the true ablation zone.

As a summary of previous studies (**Table 5**), HIFU has commonly been used for HCC as monotherapy (19–33) or in combination with other therapies, such as TACE (34–42), cryocare knife (65), and SBRT (63, 66). However, very few studies have reported the efficacy of HIFU monotherapy in patients with MLC (20, 24, 43–47) without any HIFU-based combination regimens reported. Among previous studies, high heterogeneity of the response outcome was detected. It might be caused by different response assessment criteria. We found that when using RECIST 1.1, the ORR and CR were quite low. However, ORR and CR significantly increased up to 100% by using mRECIST (**Table 5**). In our clinical practice, ablation therapies, including HIFU applied in liver cancer, often do not induce tumor shrinkage immediately postprocedure, and some even increased in volume. However, the tumors after HIFU have already been necrotic. In this situation, patients would obtain clinical benefits from HIFU and will achieve long-term survival with tumor (48, 67). Therefore, RECIST 1.1 based on the lesion volume seemed to be unsuitable for HIFU response evaluation. In our investigation, we applied both mRECIST and RECIST 1.1 (**Table 2**) criteria for tumor evaluation. Similarly, we detected great differences in remission rates between the two assessment methods. The CR rate, ORR, and DCR were estimated to be 53.8%, 66.2%, and 82.5% by mRECIST. However, when RECIST 1.1 was used, no CR was identified, and ORR and DCR were 31.3% and 71.6%, respectively, which were also significantly lower than the rates by mRECIST (*p*=0.001). The correlation between OS with RECIST 1.1 or mRECIST were also compared by us. The response status evaluated by mRECIST criteria was found to be an independent risk factor for OS in cases with both PCL and MCL. However, the results based on the RECIST 1.1 criteria did not reveal any significant correlation with OS. To sum up, we prefer mRECIST as the main criterion of efficacy evaluation in the present study, and the following discussion is mainly based on the data from mRECIST.

**Table 5:** Summary of previous data on HIFU ablation for primary and secondary liver cancer.

Study	Therapy	Patients	Response	Survival
**PLC**				
**Wu et al. (** 19 **)**	HIFU	55 Large HCC	ORR*: 52 (94.5%) CR: 2 (3.8%) PR: 50 (90.9%)	6-months OS rate: 86.1% 1-year OS rate: 61.5% 18-months OS rate: 35.3%
**Zhou et al. (** 20 **)**	HIFU	8	ORR☨: 8 (100.0%) CR: 6 (75.0%) PR: 2 (25.0%)	NA
**Zhu et al. (** 21 **)**	HIFU	16	NA	1-year OS rate: 100.0% 2-year OS rate: 83.3%
**Zhang et al. (** 22 **)**	HIFU	39 (42 lesions) HCC adjacent to major hepatic veins	ORR¶: 42 (100.0%) CR: 21 (50.0%) PR: 21 (50.0%)	1-year OS rate: 75.8% 2-year OS rate: 63.6% 3-year OS rate: 49.8% 4-year OS rate: 31.8% 5-year OS rate: 31.8%
**Zhang et al. (** 23 **)**	HIFU	6	CR¶: 6 (100.0%)	NA
**Orsi et al. (** 24 **)**	HIFU	6 Small HCC	CR☨: 6 (100.0%)	2-year OS rate: 100.0%
**Numata et al. (** 25 **)**	HIFU	21	CR^#^:18 (85.7%)	NA
**Ng et al. (** 26 **)**	HIFU	49	CR^#^: 39 (79.5%)	1-year OS rate: 87.7% 3-year OS rate: 62.4%
**Fukuda et al. (** 27 **)**	HIFU	12 Small HCC	CR^#^: 12 (100.0%)	NA
**Zhang et al. (** 28 **)**	HIFU	27 (39 lesions)	CR¶: 28 (71.8%)	NA
**Chan et al. (** 29 **)**	HIFU	27	ORR^#^: 27 (100%) CR: 23 (85.2%) PR: 4(14.8%)	1-year OS rate: 96.3% 2-year OS rate: 81.5% 3-year OS rate: 69.8%
**Cheung et al. (** 30 **)**	HIFU	100 Mixed^&^	CR^$^: 87%	NA
**Cheung et al. (** 31 **)**	HIFU	10 waitlisted for OLT	ORR^#^: 10 (100%) CR: 9 (90.0%) PR: 1 (10.0%)	NA
**Cheung et al. (** 32 **)**	HIFU	26 Recurrent HCC	ORR^#^: 15 (57.7%) CR: 13 (50%) PR: 2 (7.7%)	1-year OS rate: 84.6% 3-year OS rate: 49.2% 5-year OS rate: 32.3%
**Chok et al. (** 33 **)**	HIFU	21 waitlisted for OLT	ORR^#^: 8 (38.1%) CR: 7 (33.3%) PR: 1 (4.8%)	NA
**Ji et al. (** 48 **)**	HIFU	63	ORR^#^: 49 (77.8%) CR: 20 (31.7%) PR: 29 (46.1%)	1-year OS rate: 87.3% 2-year OS rate: 44.4%
**Wu et al. (** 34 **)**	HIFU+TACE	24	ORR*: 21 (87.5%)	MST: 11.3 months 6-month OS rate: 80.4-85.4% 1-year OS rate: 42.9%
**Li 2010 (** 35 **)**	HIFU+TACE	44 Larger HCC	ORR¶: 32(72.8%) CR: 12 (27.3%) PR: 20 (45.5%)	1-year OS rate: 72.7% 2-year OS rate: 50.0% 3-year OS rate: 31.8% 5-year OS rate: 11.4%
**Jin et al. (** 36 **)**	HIFU+TACE	73	CR^#^:33 (45.2%)	MST: 12 months 1-year OS rate: 49.1% 2-year OS rate: 18.8% 3-year OS rate: 8.4%
**Xu et al. (** 37 **)**	HIFU or HIFU +TACE/PEI	145	ORR¶: 106 (73.1%) CR: 34 (23.4%) PR: 72 (49.7%)	2-year OS rate: 46.5% (Ib) 2-year OS rate: 46.5% (IIa) 2-year OS rate: 46.5% (IIIa)
**Wang et al. (** 38 **)**	HIFU+TACE	12	ORR^#^: 10 (83.3%) CR: 10 (83.3%) PR: 0	MST: 14 months 1-year OS rate: 91.7% 2-year OS rate: 83.3%
**Zhai and Wang (** 65 **)**	HIFU+cryocare knife	40 Advanced liver cancer	ORR*: 27 (67.5%) CR: 7 (17.5%) PR: 20(50.0%)	MST: 16.4 months OS rate: 70.0%
**Wang et al. (** 66 **)**	HIFU+SBRT	76 Massive HCC	ORR^#^: 56 (73.7%) CR: 40 (52.6%) PR: 16 (21.2%)	1-year OS rate: 33.0% 3-year OS rate: 20.0% 5-year OS rate: 13.0%
**Yu et al. (** 39 **)**	HIFU+TACE	89	CR^#^: 58 (65.2%)	NA
**Ma et al. (** 63 **)**	HIFU+SBRT	96	NA	6-month OS rate: 35.3% 1-year OS rate: 10.9%
**Huang et al. (** 40 **)**	HIFU+ TACE/SonoVue	52 (73 lesions)	ORR¶: 51 (69.9%) CR: 27 (37.0%) PR: 34 (22.9%)	MST: 30-33 months 6-month OS rate: 100.0% 1-year OS rate: 89.4-95.2% 2-year OS rate: 89.3-89.4%
**Luo and Jiang (** 41 **)**	HIFU+TACE	45	ORR¶: 38 (84.4%) CR: 15 (33.3%) PR: 23 (51.1%)	NA
**Zhang et al. (** 42 **)**	HIFU+TACE	50 Middle-advanced liver cancer	ORR^#^: 45 (90.0%) CR: 20 (40.0%) PR: 25 (50.0%)	1-year OS rate: 90.0% 2-year OS rate: 80.0% 5-year OS rate: 50.0%
**MLC**				
**Zhou et al. (** 20 **)**	HIFU	4	ORR☨: 4 (100.0%) CR: 0 PR: 4 (100.0%)	NA
**Leslie et al. (** 43 **)**	HIFU	8	ORR☨: 4 (50.0%) CR: 3 (37.5%) PR: 1 (12.5%)	NA
**Park et al. (** 44 **)**	HIFU	10 (13 lesions)	ORR¶: 13 (100.0%) CR: 8 (61.5%) PR: 5 (38.5%)	NA
**Orsi et al. (** 24 **)**	HIFU	24	CR☨: 22 (91.7%)	1-year OS rate: 88.2% 2-year OS rate: 88.2%
**PLC and MLC**				
**Orgera et al. (** 45 **)**	HIFU	8 (13 lesions)	CR^#^: 11 (84.6%)	NA
**Leslie et al. (** 46 **)**	HIFU	29 Primary or metastatic	ORR^#^: 27 (93%)	NA
**Chen et al. (** 47 **)**	HIFU	187	ORR^#^: 128 (68.4%) CR: 55 (29.4%) PR: 73 (39.0%)	NA

*Response evaluation was according to RECIST 1.1 criteria.

^#^Response evaluation was according to the modified RECIST criteria.

CR was defined as total tumor necrosis, and PR was defined as patients with a necrosis area larger than 50%; or with the WHO standard.

^&^HIFU as primary treatment (n=27); as bridging therapy before OLT (n=3); recurrence of HCC after TACE (n=41); HIFU after partial hepatectomy (n=28); HIFU after OLT (n=1).

^$^The CR rate was 87% for tumor < 3 cm, with unknown number.

^†^CR was not defined.

CR, complete response; HCC, hepatocellular carcinoma; HIFU, High-intensity focused ultrasound; OLT, orthotopic liver transplantation; ORR, objective response rate; OS, overall survival; TACE, transarterial chemoembolization; PEI, percutaneous ethanol injection; PLC, primary liver cancer; PR, partial response; MLC, metastatic liver cancer; MST, median survival time; NA, not applied; SBRT, Stereotactic Body Radiation Therapy.

In consideration of the high heterogeneity between these studies, we failed to pool these results as a meta-analysis. However, we could estimate rough values of CR rate and ORR for patients with HCC and MLC who were treated with HIFU. The weighted, pooled CR rates and ORRs were 66% and 83% for HCC and 40% and 73% for MLC, respectively. Our results, CR of 63.5% and ORR of 71.8% for HCC patients and 49.5% and 63.7% in the MCL cohort, were consistent with the existing research.

We conducted subgroup analyses of the response outcomes. Patents with ECOG PS <2, LDi < 5 cm might have a better response no matter whether they are in the PLC or MLC cohorts. Furthermore, metastatic lesions from the colon and rectum also seem to have better response, which might be due to the lower degree of malignant of colorectal cancer itself.

In the present observational study, the 1-year survival rate was 70.69% (95% CI = 59.54–79.29%) in patients with HCC and 48.00% (95% CI = 40.43–55.16%) in patients with MLC. Median OS was 13 and 12 months, respectively. As a comparison, previous studies on HIFU monotherapy for cases with HCC have shown a pooled 1-year survival rate of 81.2% (range: 61.5–100.0%), which was roughly equivalent to our outcome. Only one report (24) has described the 1-year survival rate of 88.2% in 24 patients with MLC, which may be of less statistical power. Therefore, our result in MLC patients seemed to be more meaningful clinical data to reflect the impact of HIFU on the survival of MLC patients. In an intragroup comparison of our study, we found that the OS outcome of patients with PLC was superior to that of patients with MLC (**Figure 3**, *p* = 0.000). We noted that patients with MLC had worse physical status, more lesions, larger diameter, and more extrahepatic metastasis in baseline characteristics. All these may lead to a worse prognosis of MLC.

To further analyze our survival outcome, we conducted subgroup analyses. Multivariate regression analysis showed that VAS ≥ 5 and LDi ≥ 5 cm were the independent risk factors for the poor OS outcome in patients with PLC (**Figure 4** and **Supplementary Table 1**). For patients with MLC, independent risk factors were identified as VAS ≥ 5, LDi ≥ 5 cm, existence of extrahepatic metastases, and time to HIFU treatment from diagnosis < 3 months (**Figure 4** and **Supplementary Table 2**). These data have not been provided by previous studies. However, it has certain significance for the choice and prognosis of clinical patient screening and prognosis prediction.

In terms of the TEAEs, rare severe adverse reactions were observed during and after HIFU therapy. Some cases reported slightly elevated temperature, which may have been caused by the absorption heat form the necrotic tumor. These symptoms can restore itself without the need of special treatment. Diarrhea was also reported in rare cases, and we believe that it might also be caused by the water sac, which is cool to cause gastrointestinal discomfort. All these complications were not caused by nontarget sonication. Some patients before HIFU treatment felt stressful. However, after preoperative communication by informing the patient with detailed information during and after HIFU treatment, 90% of patients could control their stress in the appropriate range. Earlier research reports high-frequency HIFU-related complications, especially transient pain and superficial skin burns (81% and 39%, respectively) (46). However, our observation did not reveal any evidence of skin burn complication. The difference in HIFU equipment might cause this contrast. The HIFUNIT-9000 device applied in our center adopts a dual focus mode, which facilitates the reduction of energy on the skin during the operation. However, other types of equipment were without this design.

Several limitations should be acknowledged. (1) This is a single-center study, retrospective in nature, which has limited clinical significance. (2) HIFU ablation was conducted using a specific type of HIFU machine (HIFUINT-9000) that is different from the more commonly used integrated transducer design. The efficacy and safety could not be extended to all the HIFU equipment. (3) We did not compare HIFU with standard of care or other thermal ablation techniques, such as RFA and microwave ablation.

In conclusion, our results, which were based on an observational study of the largest sample size to date, further demonstrate the efficacy and safety of HIFU treatment for patients with primary and secondary liver cancer. HIFU might be one optimal therapy for unresectable hepatic tumor. Further well-designed RCTs are needed to evaluate the clinical efficacy of HIFU ablation, especially in combination regimens.

## Data Availability Statement

The datasets generated for this study are available on request to the corresponding author.

## Ethics Statement

The studies involving human participants were reviewed and approved by Huadong Hospital. The patients/participants provided their written informed consent to participate in this study.

## Author Contributions

HZ and YJ conceived and designed the study. YJ, JZ, LZ, YZ, and HZ collected and assembled the data. YJ and JZ performed the statistical analysis. YJ, LZ, and YZ wrote the manuscript. All authors contributed to the article and approved the submitted version.

## Conflict of Interest

The authors declare that the research was conducted in the absence of any commercial or financial relationships that could be construed as a potential conflict of interest.
