# Supplementary material for: High-Intensity Focused Ultrasound Ablation for Unresectable Primary and Metastatic Liver Cancer: Real-World Research in a Chinese Tertiary Center With 275 Cases
Source: Front Oncol. 2020 Oct 29;10:519164. doi: 10.3389/fonc.2020.519164 (PMC7658544; doi:10.3389/fonc.2020.519164)
Supplement: Supplementary file 1 [file Table_1.docx]

***Supplementary Table 1. OS outcomes and subgroup analyses of patients with primary liver cancer***

| Characteristics | MST  (95% CI) | 1-year OS  (95% CI) | Log-rank  P-value | aHR^&^ | Cox  p-value |
| --- | --- | --- | --- | --- | --- |
| Total, n=82 | 13.0 (12.5-14.0) | 70.69 (59.54-79.29) |  |  |  |
| Age  <65, n=46  ≥65, n=36 | 13.0 (12.2-14.0)  13.0 (12.3-26.0) | 67.26 (51.66-78.80)  75.00(57.46-86.13) | 0.158 | 1  1.294 (0.653-2.562) | 0.460 |
| Gender  Male, n=68  Female, n=14 | 12.8 (12.5-13.8)  13.4 (10.8-29.4) | 69.06 (56.59-78.60)  78.57 (47.25-92.54) | 0.203 | 1  2.096 (0.728-6.034) | 0.170 |
| ECOG PS  <2, n=45  ≥2, n=37 | 14.2 (13.2-26)  12.2 (9.2-12.6) | 86.61 (72.59-93.75)  51.35 (34.41-65.95) | 0.0003 | 1  1.967 (0.974-3.970) | 0.059 |
| VAS  < 5, n=50  ≥ 5, n=32 | 14.2 (13.2-26)  12.0 (8.4-12.6) | 83.95 (70.46-91.63)  50.00 (31.90-65.67) | 0.0000 | 1  2.764 (1.222-6.250) | 0.015 |
| Lesions  Single, n-16  Multiple, n=66 | 26.4 (17.2-47)  12.6 (12.2-13.2) | 93.75 (63.23-99.10)  63.57 (50.74-73.89) | 0.0000 | 1  2.649 (0.934-7.512) | 0.067 |
| LDi*  < 5, n=48  ≥ 5, n=34 | 17.2 (13.4-26.2)  10.8 (8.4-12.5) | 89.58 (76.77-95.53)  43.92 (27.04-59.59) | 0.0000 | 1  4.981 (2.184-11.360) | 0.000 |
| Protal vein invasion  Yes, n=30  No, n=52 | 12.2 (10.6-15.8)  15.3 (13.2-25.3) | 60.00 (36.32-71.18)  70.09(47.56-82.29) | 0.069 | 1  0.691 (0.594-1.282) | 0.093 |
| Tumor location  Right lobe, n=6  Left lobe, n=18  Both, n=58 | 12.0 (10.8-13.8)  14.2 (13.2-29.4)  13.4 (12.6-17.2) | 66.67 (44.73-89.31)  72.21 (52.33-83.62)  70.69 (50.74-79.33) | 0.085 | 1  0.893 (0.731-1.397)  0.977 (0.886-1.695) | 0.153 |
| Stage  Stage III, n=52  Stage IV, n-30 | 13.8 (12.5-18.0)  12.8 (10.8-13.2) | 76.92 (62.96-87.32)  59.81 (40.20-74.84) | 0.015 | 1  1.802 (0.944-3.441) | 0.074 |
| Intrahepatic metastasis status  Yes, n=18  No, n=64 | 13.8 (10.4-18.0)  12.8 (12.4-13.8) | 66.67 (40.35-83.43)  71.84 (59.09-81.23) | 0.799 | 1  1.583 (0.728-3.443) | 0.246 |
| Extrahepatic metastases status  Yes, n=20  No, n=62 | 12.8 (7.2-17.2)  13 (12.5-14.2) | 60.00 (35.73-77.60)  74.16(61.33-83.29) | 0.593 | 1  1.632 (0.776-3.435) | 0.197 |
| Histories of disease  Hepatitis [B](javascript:void(0);), n=38  Liver cirrhosis, n=19  Hypertension, n=18  Diabetes Mellitus, n=10 | 13.8 (12.4-26)  13.2 (10.4-15.5)  12.5 (10.8-14)  12.5 (3.8-14) | 70.96 (53.71-82.75)  68.42 (42.79-84.39)  68.75 (40.46-85.63)  60.00 (25.27-82.72) | 0.200^$^  0.448^$^  0.408^$^  0.439^$^ | 0.900 (0.484-1.674)  0.695 (0.340-1.419)  1.101 (0.452-2.685)  1.182 (0.371-3.762) | 0.739^$^  0.318^$^  0.832^$^  0.777^$^ |
| Prior therapies for liver cancer  Prior ≥ one therapies, n=13  Naïve, n=69 | 12.5 (6.8-13.2)  13.2 (12.5-15.5) | 53.85 (24.77-75.99)  73.88 (61.80-82.67) | 0.283 | 1  0.931 (0.432-2.006) | 0.854 |
| AFP level  Negative (< 400 μg/L), n=19  Positive (≥ 400 μg/L), n=63 | 29.4 (12.5-40.2)  12.8 (12.3-13.8) | 78.95 (53.19-91.53)  68.18 (55.14-78.16) | 0.002 | 1  1.506 (0.627-3.614) | 0.359 |
| Time to HIFU^#^  < 3, n=38  ≥ 3, n=44 | 13.2 (12.5-18.0)  12.8 (11.6-13.8) | 76.32 (59.42-86.90)  65.83 (49.84-77.80) | 0.254 | 1  0.897 (0.479-1.679) | 0.733 |
| HIFU sessions  < 5, n=14  ≥ 5, n=68 | 12.2 (6.4-14.2)  13.2 (12.6-15.5) | 57.14 (28.40-77.97)  73.47 (61.25-82.38) | 0.226 | 1  0.539 (0.237-1.225) | 0.140 |
| Indicated for RFA  Yes, n=38  No, n=44 | 13.2 (12.5-15.5)  12.8 (11.6-14.2) | 73.68 (59.67-83.32)  68.18 (53.33-77.67) | 0.179 | 1  1.424 (0.599-4.025) | 0.241 |
| Response status by mRECIST  Responser, n=60  Non-responser, n=22 | 17.2 (15.5-26.2)  12.0 (8.9-12.9) | 76.67 (67.67-92.36)  54.54 (37.40-62.13) | 0.023 | 1  1.725 (1.173-2.639) | 0.037 |
| Response status by RECIST1.1  Responser, n=26  Non-responser, n=56 | 13.8 (12.5-18.0)  12.5 (10.8-14.2) | 73.08 (62.77-89.63)  69.64 (51.32-80.64) | 0.257 | 1  1.377 (0.897-2.044) | 0.074 |

* LDis for the patients with multiple lesions were the sum of the longest diameter of all these lesions.

^#^ Time to HIFU ablation from the diagnosis of HCC.

^&^ aHRs were adjusted by variables including ECOG PS, VAS, lesion number, LDi, stage, and AFP levels.

^$^ These patients were compared with the cases without hepatitis [B](javascript:void(0);), liver cirrhosis, hypertension, or diabetes mellitus, respectively.

**Abbreviation**: AFP, alpha-fetoprotein; DCR, disease control rate; ECOG PS, Eastern Cooperative Oncology Group Performance Status; HCC, Hepatocellular Carcinoma; HIFU, High-Intensity Focused Ultrasound; LDi, longest diameter; MST, median survival time; PLC, primary liver cancer; VAS, visual analogue scale.

***Supplementary Table 2. OS outcomes and subgroup analyses of patients with metastatic liver cancer***

| Characteristics | | MST  95% CI | 1-year OS  95% CI | Log-rank  P-value | aHR^&^ | | | Cox  p-value |
| --- | --- | --- | --- | --- | --- | --- | --- | --- |
| Total, n=175 | | 12.0 (9.5-12.4) | 48.00 (40.43-55.16) |  |  | | |  |
| Age  <65, n=94  ≥65, n=81 | | 12.2 (9.6-12.6)  10.2 (7.5-12.2) | 53.19 (42.64-62.66)  41.98 (31.16-52.40) | 0.223 | 1  1.219 (0.921-2.160) | | | 0.109 |
| Gender  Male, n=102  Female, n=73 | | 12.4 (10.0-12.8)  9.4 (7.2-12.1) | 53.92 (43.79-63.01)  39.73 (28.54-50.67) | 0.027 | 1  1.127 (0.759-1.672) | | | 0.552 |
| ECOG PS  <2, n=67  ≥2, n=108 | | 13.2 (12.4-14.0)  9.0 (7.2-11.5) | 70.15 (57.65-79.60)  34.26 (25.48-43.20) | 0.002 | 1  1.299(0.940-2.245) | | | 0.140 |
| VAS  < 5, n=164  ≥ 5, n=11 | | 12.0 (9.8-12.4)  7.2 (4.5-12.6) | 49.39 (41.53-56.76)  27.27 (6.52-53.89) | 0.033 | 1  2.360 (1.182-4.710) | | | 0.015 |
| Lesions  Single, n=27  Multiple, n=148 | | 12.5 (6.8-17.2)  11.5 (9.2-12.4) | 55.56 (35.22-71.81)  46.62 (38.42-54.40) | 0.037 | 1  1.540 (0.858-2.764) | | | 0.148 |
| LDi*  < 5, n=84  ≥ 5, n=91 | | 12.4 (11.5-13.0)  8.8 (7.0-12.1) | 55.95 (44.71-65.78)  40.66 (30.55-50.51) | 0.0001 | 1  1.692 (1.178-2.431) | | | 0.004 |
| Protal vein invasion  Yes, n=37  No, n=138 | | 10.2 (9.2-12.1)  12.5 (9.5-17.2) | 45.95 (39.97-63.21)  48.56 (40.44-64.65) | 0.069 | 1  0.718 (0.634-1.312) | | | 0.137 |
| Tumor location  Right lobe, n=10  Left lobe, n=21  Both, n=144 | | 12.0 (10.8-13.8)  14.2 (13.2-29.4)  13.4 (12.6-17.2) | 40.00 (22.73-64.13)  47.62 (35.45-72.02)  48.61 (36.39-59.97) | 0.085 | 1  0.748 (0.691-1.971)  0.827 (0.646-2.357) | | | 0.293 |
| Primary tumor site for LM  Colon, n (%), n=39  Pancreas, n (%), n=40  Gastric, n (%), n=27  Rectum, n (%), n=15  Breast, n (%), n=16  Gallbladder, n(%),n =8  Others^☨^,n =30 | | 13.2 (12.0-15.8)  7.2 (6.2-9.5)  12.4 (9.0-14.2)  13.2 (12.4-14.0)  7.0 (4.2-12.8)  6.8 (4.5-12.4)  12.0 (7.4-13.2) | 66.67 (49.61-79.09)  15.00 (6.09-27.64)  59.26 (38.63-74.99)  86.67 (56.39-96.49)  37.50 (15.42-59.77)  25.00 (3.71-55.81)  50.00 (31.30-66.12) | 0.023^  0.001^  0.076^  0.044^  0.063^  0.099^  0.738^ | 0.886 (0.708-1.071)  1.277 (1.045-1.652)  0.904 (0.746-1.124)  0.746 (0.529-1.277)  1.366 (0.979-2.018)  1.344 (0.623-3.279)  1.034 (0.879-1.427) | | | 0.095^  0.037^  0.632^  0.429^  0.088^  0.756^  0.912^ |
| Extrahepatic metastases status  No, n=113  Yes, n=62 | | 12.4 (11.6-12.6)  8.2 (6.2-10.5) | 56.64 (47.00-65.18)  32.26 (21.09-43.93) | 0.0004 | 1  1.596 (1.109-2.296) | | | 0.012 |
| Histories of disease  [Hepatitis](javascript:void(0);) [B](javascript:void(0);), n=6  Liver cirrhosis, n=1  Hypertension, n=28  Diabetes Mellitus, n=16 | | 12.4 (3.4-NE)  7.0 (NE-NE)  12.2 (8.8-15.0)  9.5 (4.8-13.2) | 66.67 (19.46-90.44)  0.0 (NE-NE)  57.14 (37.06-72.95)  50.00 (24.52-71.05) | 0.460^$^  0.300^$^  0.206^$^  0.413^$^ | 1.051 (0.391-2.825)  2.226 (0.231-21.459)  0.813 (0.489-1.355)  1.251 (0.675-2.319) | | | 0.922^$^  0.489^$^  0.428^$^  0.478^$^ |
| Prior therapies for liver cancer  Naïve, n=169  Prior ≥ one therapies, n=6 | | 12.0 (9.4-12.4)  12.4 (6.8-NE) | 47.34 (39.65-54.63)  66.67 (19.46-90.44) | 0.443 | 1  0.982 (0.898-1.073) | | | 0.690 |
| AFP level  Negative (< 400 μg/L), n=151  Positive (≥ 400 μg/L), n=24 | | 11.5 (9.2-12.2)  12.5 (6.8-13.5) | 45.70 (37.61-53.41)  62.50 (40.30-78.42) | 0.460 | 1  0.817 (0.477-1.402) | | | 0.464 |
| TTM  Synchronous, n=57  Metachronous(< 1 year) , n=71  Metachronous (≥ 1 year), n=47 | | 9.4 (7.2-12.1)  12.4 (10-12.6)  12.0 (7.2-12.4) | 38.60 (26.11-50.92)  54.93 (42.67-65.61)  48.94 (34.12-62.19) | 0.706 | 1  0.969 (0.762-1.233) | | | 0.800 |
| Time to HIFU^#^  < 3, n=88  ≥ 3, n=87 | | 12.2 (9.8-13.0)  10.0 (7.0-12.2) | 51.14 (40.28-60.98)  44.83 (34.20-54.88) | 0.0003 | 1  1.590(1.106-2.286) | | | 0.012 |
| HIFU sessions  < 5, n=47  ≥ 5, n=143 | | 7.2 (6.0-10.5)  12.2 (10.4-12.5) | 34.09 (20.67-47.97)  52.67 (43.79-60.79) | 0.025 | 1  0.710 (0.462-1.089) | | | 0.116 |
| Indicated for RFA  Yes, n=74  No, n=101 | 12.2 (10.0-15.0)  12.0 (10.4-13.2) | | 45.95 (32.83-64.76)  39.60 (23.93-56.65) | 0.095 | | 1  1.693 (0.644-3.014) | 0.197 | |
| Response status by mRECIST  Responser, n=118  Non-responser, n=57 | 12.4 (10.4-17.2)  8.8 (6.8-10.0) | | 52.54 (45.67-62.33)  38.60 (29.60-43.98) | 0.033 | | 1  1.350 (1.083-2.350) | 0.014 | |
| Response status by RECIST1.1  Responser, n=51  Non-responser, n=124 | 11.5 (9.2-13.5)  9.4 (7.2-10.4) | | 50.98 (42.26-59.33)  46.77 (40.79-50.32) | 0.098 | | 1  1.106 (0.770-1.937) | 0.109 | |

* LDis for the patients with multiple lesions were the sum of the longest diameter of all these lesions.

^#^ Time to HIFU ablation from the diagnosis of HCC.

^&^ aHRs were adjusted by variables including gender, ECOG PS, VAS, Lesion number, LDi, extrahepatic metastases status, time to HIFU from diagnosis, and HIFU sessions.

^ Each subgroup was compared with the overall cohort without this subgroup itself.

^$^ These patients were compared with the cases without hepatitis [B](javascript:void(0);), liver cirrhosis, hypertension, or diabetes mellitus, respectively.

**Abbreviation**: AFP, alpha-fetoprotein; DCR, disease control rate; ECOG PS, Eastern Cooperative Oncology Group Performance Status; HCC, Hepatocellular Carcinoma; HIFU, High-Intensity Focused Ultrasound; LDi, longest diameter; MST, median survival time; NE, not estimated; PLC, primary liver cancer; VAS, visual analogue scale.
